# Multiple directional DWI combined with T2WI in predicting muscle layer and Ki‐67 correlation in bladder cancer in 3.0‐T MRI

**DOI:** 10.1002/cam4.5782

**Published:** 2023-03-14

**Authors:** Wei Zhang, Zhichao Zhang, Weixiong Xiao, Yiqian Wang, Liefu Ye, Yongbao Wei, Min Luo

**Affiliations:** ^1^ Shengli Clinical Medical College of Fujian Medical University Fuzhou China; ^2^ Department of Radiology Fujian Provincial Hospital Fuzhou China; ^3^ Department of Radiology Fujian Medical University Cancer Hospital Fujian Cancer Hospital Fuzhou China; ^4^ Department of Urology Fujian Provincial Hospital Fuzhou China

**Keywords:** bladder cancer, diffusion‐weighted imaging, magnetic resonance imaging, neoplasm staging

## Abstract

**Objective:**

To investigate the value of 3.0T MRI multi‐directional diffusion‐weighted imaging (DWI) combined with T2WI morphological features and lesion distribution in preoperative prediction of muscle layer invasion of bladder cancer (BC) and the correlation with postoperative Ki‐67.

**Materials and Methods:**

This retrospective study enrolled patients with BC between 2019 and 2021. Patients with muscular invasive bladder cancer (MIBC) or non‐muscular invasive BC (NMIBC) were also analyzed by preoperative 3.0T MRI aFostic efficacy.

**Results:**

A total of 186 patients were enrolled. About 27 patients with MIBC (35 lesions in total) and 62 with NMIBC (99 lesions in total). We found the tumor with a larger size, a wide base, and a smaller apparent dispersion coefficient (ADC) value and normalized ADC(nADC) value, without a stalk, presenting a greater risk of muscle invasion. ADC value, nADC value, maximum diameter, and stalk were independently associated with muscle invasion. Lesions located at the bladder fundus or involvement of multiple sites were independently associated with muscle invasion compared to the bladder body. In combination with morphological features, the AUCs of ADC and nADC showed accuracies of 0.925 and 0.947–0.951, respectively. TADC and nTADC showed the best diagnostic efficacy in multiple respects. KI‐67 LI was negatively correlated with ADC and nADC values.

**Conclusions:**

This is the first report in which we found Multi‐directional DWI combined with T2WI in 3.0T MRI can be used to predict the muscle layer invasion of bladder cancer. ADC values reflect the muscular invasion of bladder cancer and show a moderate negative correlation with Ki‐67. It is especially suitable for bladder cancer patients with renal insufficiency or tumor recurrence.

## Graphical abstract

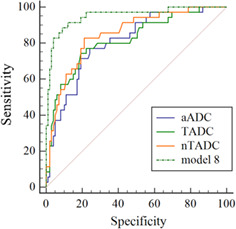

## 1 INTRODUCTION

Bladder cancer is the most common urinary tract tumor, with approximately 573,000 new cases and 213,000 deaths per year, ranking tenth in the diagnosis of cancer diseases in the global population and rising to seventh in the male population, and the ratio of male‐to‐female incidence is between 4: 1.^1^ Up to now, the etiology of bladder cancer is not fully understood. Still, it is thought to be related to specific genetic mutations. It is generally accepted that smoking, prolonged exposure to aromatic types of work and prolonged local irritation of the bladder mucosa are predisposing, especially smoking.^1^, ^2^, ^3^

Bladder cancer (BC) is clinically classified as non‐muscle invasive bladder cancer (NMIBC) and muscle‐invasive bladder cancer (MIBC), according to the European Association of Urology (EAU) guidelines. Currently, transurethral resection of bladder tumor (TURBT) is mainly used to obtain pathological specimens for staging. However, TURBT also has some limitations. The study results show that approximately 25% of the specimens based on TRUBT misdiagnose MIBC as NMIBC, resulting in lower bladder cancer staging.^4^ Inadequate staging of muscle invasion will affect the treatment plan, as most NMIBC are treated by TURBT, while MIBC is treated by radical cystectomy combined with adjuvant therapy. For a more accurate staging of bladder cancer, a second TURBT is recommended.^5^, ^6^ Accurate preoperative diagnosis of muscle invasion in bladder cancer is important in the appropriate treatment choice.

Imaging has played an indelible role in diagnosing and evaluating bladder cancer, especially with multiparametric MRI (mpMRI) development. In 2018, the Vesical Imaging‐Reporting and Data System (VI‐RADS) made staging more clinically relevant.^4^, ^7^ However, in some patients with severe renal insufficiency or gadolinium hypersensitivity, dynamic contrast enhancement (DCE) was not performed, which would affect the VI‐RADS score regarding blood supply and muscle infiltration. In addition, the gadolinium contrast agent may induce mitochondrial toxicity, cell death, and the occurrence of nephrogenic systemic fibrosis (NFS) in human neurons.^8^, ^9^ Therefore, the search for a safe and accurate pre‐scholarly assessment of imaging has become a clinical need. Furthermore, VI‐RADS only quantitative indicator in the scoring system (whether the lesion is larger than 1 cm) is used to classify scores of 1 and 2, but the likelihood of muscle infiltration is small for scores of 2 and below; for lesions of 3 and above, we need some quantitative indicators that are more intuitive and easier to measure for risk stratification. Diffusion‐weighted imaging (DWI) is a non‐invasive imaging technique that does not require contrast and quantifies the diffusion of water molecules in tissue by the apparent dispersion coefficient (ADC). Previous studies have shown that ADC values have good diagnostic efficacy in tumor benignity and malignancy and monitoring drug efficacy.^10^, ^11^

In recent years, ADC values have also been reported to have good predictive value for BC muscle infiltration, but most are based on axial ADC measurements.^12^, ^13^, ^14^ BC is a highly heterogeneous tumor, and the molecular subtypes may differ in different parts of the same tumor tissue,^15^ resulting in different growth rates and patterns in each direction, and simple axial measurements only represent the largest level of the tumor. The measurement of purely axial position only represents the degree of tumor cell dispersion at the largest level and cannot reflect the anisotropy of the tumor. When BC is mainly growing up and down, the sagittal or coronal view can show whether the BC is stalked and invades the muscle layer, and the integrated three‐directional view can show the tumor morphology in a panoramic view, better avoiding the necrotic and cystic areas, and make the ADC measurement more closely match the tumor cell components of BC. Therefore, this paper aims to use three‐directional DWI combined with high‐resolution T2WI imaging for preoperative staging of BC muscle layer invasion and to investigate the correlation with Ki67 to provide a reference for clinicians in selecting treatment strategies and prognostic assessment.

## 2 MATERIALS AND METHODS

### 2.1 General information

**FIGURE 1:**
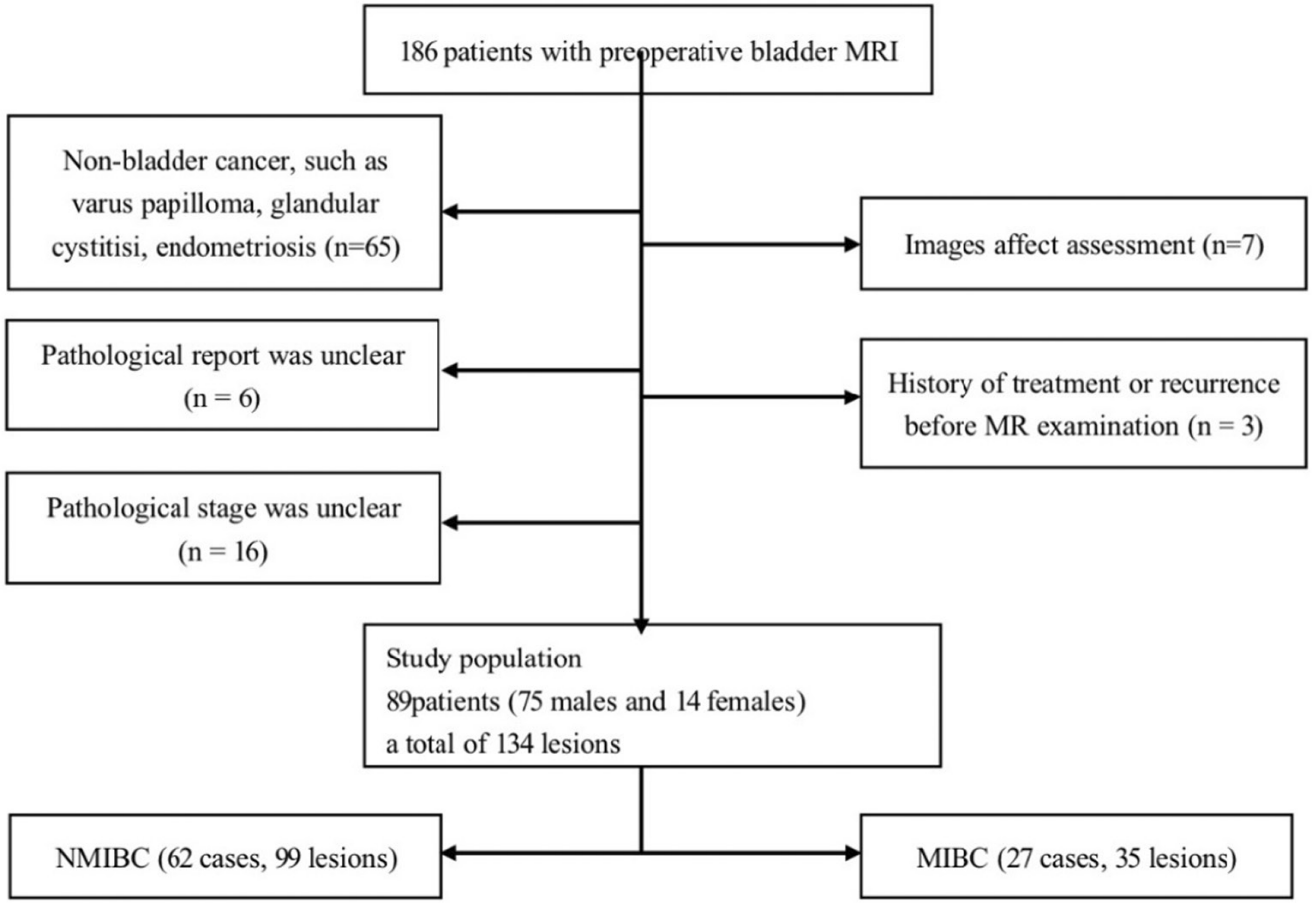
Flowchart of selection of study population. MRI, magnetic resonance imaging.

This retrospective study was approved by the Institutional Review Board, with informed consent waived. Patients with clinically suspected bladder cancer were collected between October 2019 and June 2021 based on the following inclusion criteria: (a) Bladder mass was found by ultrasound or CT examination; (b) Before or 14 days after cystoscopy; (c) Scanning on the same Prisma 3.0T MRI, the maximum diameter of all lesions was ≥0.5 cm. The exclusion criteria were as follows: (a) Postoperative pathological part of bladder cancer, such as inverted papilloma, glandular cystitis, endometriosis, etc.; (b) Insufficient bladder filling, motion artifacts, distortion in images affect assessment; (c) Postoperative pathology did not include muscle layer, the pathological stage was not precise; (d) In multiple lesions, the location of the pathological report was unclear; (e) History of treatment or recurrence before MR examination. The process of study population selection is depicted in Figure 1.

### 2.2 Preparation for mp‐MRI

Patients should empty their bladder 30 min to an hour before the examination and drink 500–750 mL water to scan when the bladder is optimally full. If the bladder is overfilled, appropriate urine may be expelled before the examination.

### 2.3 Magnetic resonance imaging protocol

The MRI was performed in the supine position using an 18‐channel volumetric coil and a 32‐channel integrated spine coil of a 3.0T MRI system (magnetom Prisma; Siemens Healthcare, Erlangen, Germany). The acquired MR images included T2‐weighted images (T2WI) and diffusion‐weighted images (DWI) in three planes: maximal axial, oblique coronal, and sagittal (Table S1).

### 2.4 Magnetic resonance image analysis

The imaging assessment for eligible cases was performed independently by two radiologists on a Siemens Syngo through the MR workspace, with 15 and 5 years of experience, respectively. These radiologists did not participate in the case selection process to avoid potential bias and were blinded to the pathology reports. The reading order was randomized for each reader. For each predetermined index lesion, the readers evaluated the basal, stalk, number, and maximum diameter on T2WI. And the differences in opinion were resolved by consensus. According to the angle between the base of the lesion and the bladder wall, an angle ≥90° was considered a wide base and less than 90° was considered a narrow base. The maximum diameter of the tumor was selected in three directions and averaged between two readers. MIBC on T2WI was defined as a tumor with moderate signal invasion into the muscular layer of the bladder, resulting in a low signal discontinuity in the muscular layer, or invasion of the muscular layer into the surrounding fat space and adjacent tissues. DWI sequences showed high signal tumor tissue invading the muscular layer of the bladder or extending into the bladder surrounding fat space and adjacent tissues, and the corresponding ADC images showed a low signal. The region of interest (ROI) was drawn within the ADC map lesion in three directions. The ROI size of the lesion was selected based on the following principles: DWI combined with T2WI images to determine the maximum level of the lesion in three directions, and the ROI was outlined manually along the tumor edge, avoiding tumor necrosis, hemorrhage and stalk. Axial ADC values (aADC values), coronal ADC values (cADC values), sagittal ADC values (sADC values), and three‐dimensional mean values (TADC values) were taken for analysis. TADC values = (aADC + cADC + sADC)/3. The right iliac muscle ADC value was measured simultaneously; the ROI was taken as a circle, 50–70 mm^2^, and the measurements were averaged three times. The normalized ADC value rate (nADC value) = ADC value/iliac ADC value (Figure S1).

### 2.5 Cancer staging and pathological analysis

According to the European Association of Urology (EAU) guidelines, NMIBC includes carcinoma in situ(Tis), non‐invasive papillary carcinoma (Ta), and bladder urothelial carcinoma (T1) limited to mucosa and submucosa; MIBC includes invasion of the muscular layer of the bladder wall to a depth of less than 1/2 (T2a), invasion of more than 1/2 of the muscular layer but not reaching the plasma layer of the bladder wall (T2b), invasion of adipose tissue of the outside of the bladder without invading the surrounding organs (T3), and invasion of surrounding organs (T4). In addition, papillary urothelial carcinoma with low malignant potential, low‐grade urothelial carcinoma, and high‐grade urothelial carcinoma were classified according to the degree of differentiation. All specimens were fixed in 10% neutral formalin, routinely dehydrated, paraffin‐embedded, serially sectioned at 4 μm thickness, HE stained, and observed by light microscopy. Immunohistochemical staining was performed by the EliVision two‐step method, and Ki67 kits were purchased from Fuzhou Maixin Company, and the specific steps were performed strictly according to the kit instructions. Ki‐67 positive was defined as the appearance of transparent brownish‐yellow granules in the nuclei of cancer cells, and the proportion of positive cells was counted by taking three representative 100 × high magnification fields.

### 2.6 Statistical analysis

SPSS 25.0 and MedCalc 19.0.7 software were used for statistical analysis. Cohen's κ statistics for qualitative variables and intraclass correlation coefficients (ICC) for quantitative variables were used to evaluate inter‐reader agreement, and κ and ICC values >0.75 indicated good agreement. Univariate factors were analyzed using the chi‐square test, exact test, *t*‐test, and Mann–Whitney U rank sum test. Statistically significant variables were included in a dichotomous logistic regression equation to analyze the risk factors associated with BC muscle infiltration. The ROC curve was used to analyze the diagnostic efficacy, and the maximum Youden index was used to obtain the optimal diagnostic threshold. Sensitivity, specificity, and accuracy were calculated; a *p* < 0.05 indicated that the difference was statistically significant.

## 3 RESULTS

### 3.1 Basic clinical characteristics of BC patients

A total of 186 patients were enrolled. Finally, 89 patients (75 males and 14 females, a total of 134 lesions; the average age was 62.2 years; age range, 16–92 years) were enrolled in our retrospective study. The clinical manifestations of patients were hematuria, with or without frequent urination, urgency of urination, and pain of urination. According to muscle layer invasion, BC was divided into MIBC (27 cases, 35 lesions) and NMIBC (62 cases, 99 lesions). If the lesions were multiple and contained both NMIBC and MIBC, the patients were classified as MIBC for clinical data analysis (Figure 1). There was no significant difference in gender between 27 patients with MIBC (35 lesions) and 62 patients with NMIBC (99 lesions) (*p* > 0.05). The risk of muscle infiltration of bladder cancer increases with age (*p* < 0.05). Patients with MIBC had more hematuria than patients with NMIBC, and the difference was statistically significant (*p* < 0.05) (Table 1).

**TABLE 1:** Characteristics of 89 patients with bladder cancer.

	Gender (*n* = 89)		Age	Hematuria (*n* = 89)		
	Male	Female		No	Occult blood	Gross hematuria
NMIBC	52	10	61.0 (53.0–69.0)	13	8	41
MIBC	23	4	68.0 (56.0–75.0)	0	6	21
*x* ^*2*^ */Z*	0.025		−1.982	8.130		
*p*	0.876		0.047	0.018		

### 3.2 Basic characteristics of lesions, ADC values and normalized ADC values in BC patients

**FIGURE 2:**
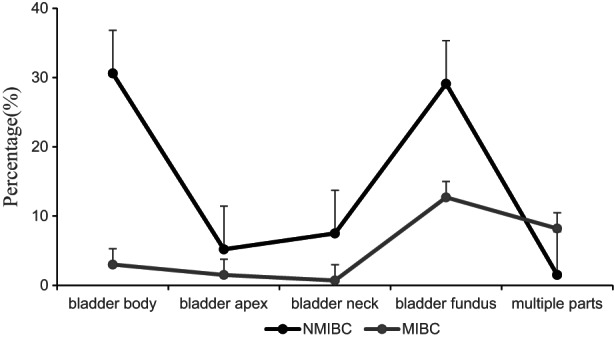
Sites of bladder cancer.

**FIGURE 3:**
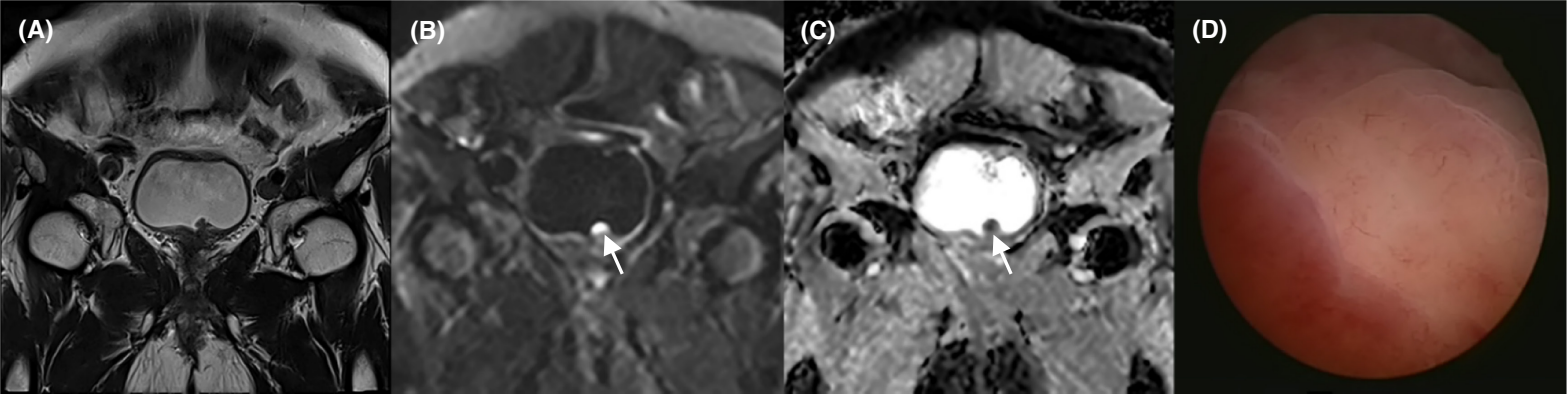
Images in a patient with an exophytic tumor located at the bladder neck. (A) Coronal T2‐weighted images shows an exophytic tumor with a low signal at the bladder neck. (B) Diffusion‐weighted imaging shows a significantly high signal (arrow). (C) The apparent diffusion coefficient map shows a low signal (arrow). (D) Cystoscopy shows an elevated mass with lobulated margins.

**FIGURE 4:**
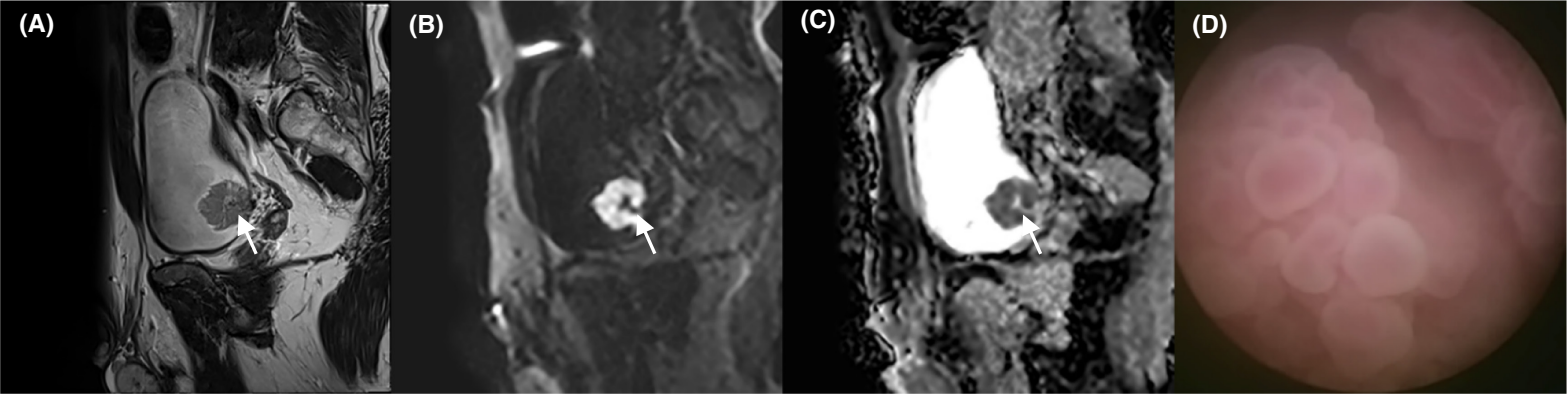
Images in a patient with an exophytic tumor located at the bladder body. (A) Sagittal T2‐weighted images shows an exophytic tumor with a low‐signal intensity stalk (arrow). (B) Diffusion‐weighted imaging shows a significantly high signal of the tumor with a low signal of its stalk (arrow). (C) The apparent diffusion coefficient map shows the low signal of the tumor with a high signal of its stalk (arrow). (D) Cystoscopy shows a cauliflower‐shaped mass with clear borders and a stalk on the right wall of the bladder, 0.5 cm from the right ureter.

**FIGURE 5:**
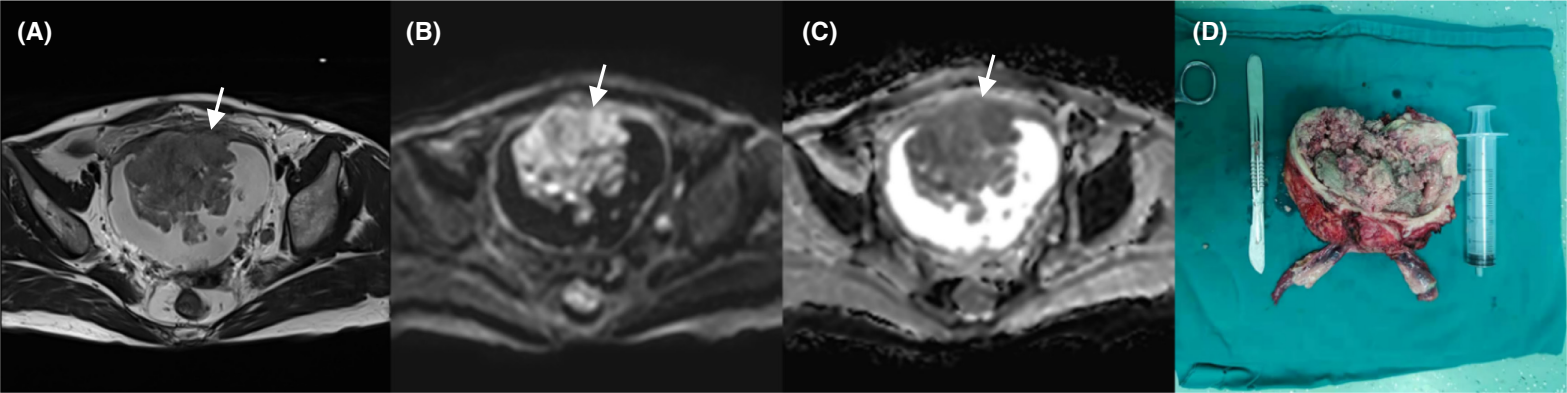
Images in a patient with an exophytic tumor located at the bladder apex. (A) The Axial T2‐weighted image shows an exophytic tumor with irregular margins and invasion of the muscle layer (arrow). (B) Diffusion‐weighted imaging shows a significantly high signal of the tumor and the high signal is up to the bladder muscle layer (arrow). (C) The apparent diffusion coefficient map shows a low signal of the tumor. (D) The surgical specimen shows a large cauliflower‐shaped mass in the bladder cavity.

The location of NMIBC and MIBC is shown in Figure 2. BC was prone to occur in the trigonometric region of the bladder's fundus and the bladder's body (47.46% and 38.14%). MIBC versus NMIBC at the fundus of bladder (12.68% vs. 29.10%), MIBC versus NMIBC in bladder body (2.99% vs. 30.60%). If a single lesion invades multiple parts simultaneously, the MIBC versus NMIBC was (8.21% vs. 1.49%). In addition, in 134 lesions, lesions with no stalk or wide base were more likely to have muscle infiltration. The risk of muscle infiltration was greater when the maximum diameter of the lesion was longer and the ADC value and the normalized ADC value were lower (*p* < 0.05). (Tables 2, 3 and Figures 3, 4, 5).

**TABLE 2:** Morphological characteristics of bladder cancer lesions.

	Number of patients (*n* = 89)		Stalk (*n* = 134)		Base (*n* = 134)		Maximum diameter (cm)
	Single	Multiple	Yes	No	Narrow	Wide	
NMIBC	40	22	60	39	53	46	1.5 (1.1–2.4)
MIBC	13	14	9	26	6	29	3.6 (2.6–5.0)
*x* ^*2*^ */Z*	2.092		12.604		13.897		−6.480
*p*	0.148		<0.001		<0.001		<0.001

**TABLE 3:** ADC values (×10^−3^ mm^2^/s) and nADC values in different directions of NMIBC and MIBC lesions.

	aADC	cADC	sADC	TADC	naADC	ncADC	nsADC	nTADC
NMIBC	1.118 ± 0.244	1.135 ± 0.242	1.118 ± 0.228	1.1241 ± 0.23	0.670 ± 0.156	0.680 ± 0.153	0.670 ± 0.146	0.673 ± 0.148
MIBC	0.860 ± 0.182	0.861 ± 0.214	0.850 ± 0.21	0.8576 ± 0.19	0.497 ± 0.988	0.498 ± 0.115	0.490 ± 0.114	0.495 ± 0.106
*t*	5.715	5.919	6.073	6.089	7.561	7.346	6.582	7.677
*p*	<0.001	<0.001	<0.001	<0.001	<0.001	<0.001	<0.001	<0.001

### 3.3 Prediction of muscle layer invasion

As the tumor ADC and nADC had a pairwise correlation coefficient above 0.80, to avoid multicollinearity problems, they were not inputted into the multivariable logistic regression analysis simultaneously. Regression analysis showed that ADC value (OR 0.009–0.014), nADC value (OR <0.001), maximum diameter (OR 2–2.156), and stalk (OR 14.741–26.010) were independently associated with muscle invasion. The bladder fundus (OR 7.829–12.215) or involvement of multiple sites (OR 21.887–37.506) were independently associated with muscle invasion compared to the bladder body. (*p* < 0.05) (Table 4).

**TABLE 4:** Preoperative ADC value and nADC value combined with MRI morphological characteristics and distribution in predicting the muscular invasive of bladder cancer.

	Model 1		Model 2		Model 3		Model 4		Model 5		Model 6		Model 7		Model 8	
	OR	*p*	OR	*p*	OR	*p*	OR	*p*	OR	*p*	OR	*p*	OR	*p*	OR	*p*
ADC value																
Axial	0.011	0.016														
Cor			0.014	0.014												
SAG					0.012	0.013										
Three‐dimensional		·					0.009	0.012								
nADC value																
Axial									<0.001	0.004						
Cor											<0.001	0.004				
Sag													<0.001	0.003		
Three‐dimensional															<0.001	0.003
Maximum diameter	2.119	0.010	2.156	0.008	2.138	0.008	2.081	0.012	2.015	0.017	2.092	0.011	2.076	0.012	2.000	0.018
Base	1.911	0.387	2.219	0.285	2.133	0.304	2.024	0.584	1.440	0.644	1.756	0.474	1.666	0.508	1.545	0.579
Stalk	19.072	<0.001	14.741	<0.001	14.853	<0.001	16.205	<0.001	26.010	<0.001	18.382	<0.001	18.692	<0.001	21.330	<0.001
Location																
Bladder body	Reference		Reference		Reference		Reference		Reference		Reference		Reference		Reference	
Bladder apex	3.845	0.376	3.796	0.377	3.854	0.373	3.920	0.368	4.627	0.317	4.173	0.347	4.324	0.338	4.505	0.325
Bladder neck	2.138	0.618	1.614	0.757	1.568	0.775	1.674	0.741	1.579	0.773	1.100	0.953	1.013	0.994	1.103	0.952
Bladder fundus	9.701	0.007	7.829	0.009	8.519	0.010	8.962	0.008	12.215	0.005	8.756	0.008	9.639	0.009	10.563	0.007
Multiple parts	22.018	0.029	37.506	0.016	27.579	0.020	30.638	0.018	21.887	0.027	35.898	0.013	25.219	0.019	29.145	0.016

*Note*: The C‐index of model 1 was 0.936 (95% CI: 0.885–0.986); The C‐index of model 2 was 0.941 (95% CI: 0.894–0.988); The C‐index of model 3 was 0.937 (95% CI: 0.885–0.989); The C‐index of model 4 was 0.939 (95% CI: 0.890–0.988); The C‐index of model 5 was 0.947 (95% CI: 0.0.901–0.992); The C‐index of model 6 was 0.949 (95% CI: 0.906–0.992); The C‐index of model 7 was 0.948 (95% CI: 0.901–0.994); The C‐index of model 8 was 0.951 (95% CI: 0.907–0.994).

Abbreviations: OR, odds ratio; *p*, *p* value.

**FIGURE 6:**
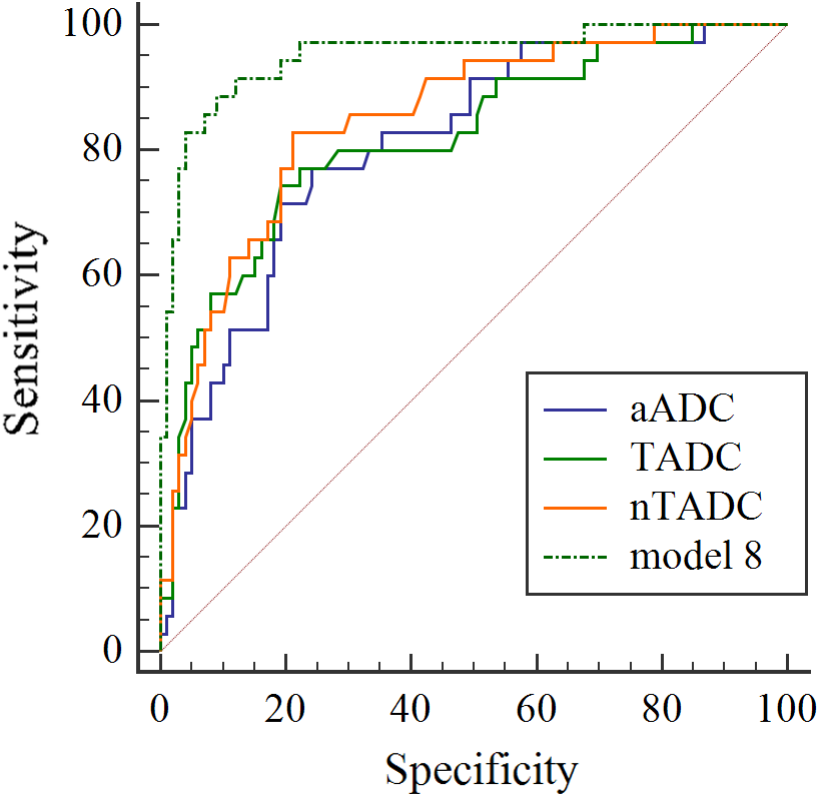
ROC curve representing aADC, TADC, nTADC, model 8 in discriminating NMIBC from MIBC. For Axial ADC values, the area under the ROC curve (AUC) was 0.808 with an accuracy of 0.761. TADC values showed an AUC of 0.817 with an accuracy of 0.791. nTADC showed an AUC of 0.849 with an accuracy of 0.799. Combined with morphological features, nTADC showed an AUC of 0.951 with an accuracy of 0.903.

The ROC analysis of tumor ADC values in different directions AUC was 0.808–0.812, and the TADC value AUC was 0.817. The normalized ADC values in different directions AUC was 0.832–0.840, and the nTADC value AUC was 0.849. The results suggest that TADC value has higher diagnostic efficacy for BC muscle infiltration than single‐direction ADC values, but the difference was not statistically significant by DeLong text. The same results are obtained in the normalized ADC values. However, the AUC values of nTADC were statistically different from the AUC values of aADC and sADC. The AUC for models 1–4 was 0.936–0.941, and the AUC for model 5–9 was 0.947–0.951. The results suggest that ADC values and normalized ADC values in combination with lesion morphology and location would further improve diagnostic efficacy (Figure 6).

### 3.4 Postoperative pathological differentiation and the correlation between Ki67 and ADC and nADC

According to the degree of differentiation, the BC were classified as a papillary urothelial neoplasm of low malignant potential (16 cases, 16 lesions), low‐grade papillary urothelial carcinoma (37 cases, 60 lesions), and high‐grade papillary urothelial carcinoma (36 cases, 58 lesions). The lower the degree of differentiation, the greater the risk of muscle infiltration (*x* ^2^ = 26.906, *p* < 0.001). The Ki67 LI (%) was 20 (10, 30) and 60 (30, 80) for NMIBC and MIBC, respectively. It was moderately negatively correlated with ADC values and nADC values, with correlation coefficients of 0.604–0.627 and 0.608–0.634, respectively.

### 3.5 Inter‐reader agreement

There was good agreement among the radiologists in assessing the tumor base, stalk, and site, with a κ range of 0.80–0.95. Among them, there was almost perfect agreement on whether the tumor was stalked (κ, 0.95). There was good agreement in measuring quantitative information iliac muscle ADC values, ADC values in different orientations, and maximum tumor diameter, with an ICC range of 0.81–0.95.

## 4 DISCUSSION

Accurate preoperative diagnosis of muscle invasion in bladder cancer is vital in the appropriate treatment choice. Although TURBT is used as a definitive treatment for most NMIBC and serves as a diagnostic procedure for most MIBC, it has been shown that the staging grade of underestimated BC exists in about 25%–50% of patients.^4^, ^16^, ^17^ For a more accurate staging of bladder cancer, a second TURBT is recommended.^5^ If a useful quantitative indicator is found, it is expected that these TURBTs may be unnecessary.

In our study, ADC was an excellent predictor of muscle infiltration in bladder cancer. The AUCs of ADC values measured between MIBC and NMIBC were 0.808–0.817. These findings are in agreement with that of prior research.^12^, ^13^, ^18^ Our study also showed no difference in the AUC values of the bladder tumor between the three ROI positioning methods and TADC in the assessment of BC muscle invasion. Similar results were reported in the study by Hongyi Li et al.^19^ The reason may be that after avoiding necrosis, cystic areas, and stalk, the tumor is essentially homogeneous in the degree of diffusion. The AUCs of ADC values for predicting muscle invasion in different directions were 0.808, 0.812, and 0.811, respectively, while the AUC under the TADC curve measured in three directions was 0.817. Although no significant differences were found between each AUC, the results of our study show that TADC may be superior to the single‐direction ADC values for assessing muscle invasion of BC. To further improve the diagnostic performance of ADC values and to reduce the diagnostic error between different MRI scanners, some studies used the ratio of ADC values of lesions to ADC values of controls to derive nADC.^20^, ^21^ Since the iliacus muscle was easily accessible in the images of BC and its internal fatty degeneration or atrophy was less common than that of the gluteus muscle, using this as a reference to normalize the ADC values of BC was a more objective and rigorous way to reduce the subjective errors in measurement. The AUC of nADC for predicting muscle invasion in different directions was 0.832–0.840, while the AUC increased to 0.849 under the nTADC curve, all superior to ADC. And the AUC values of nTADC were statistically different from the AUC values of aADC and sADC. The reasons may be nADC overcomes ADC variability attributable to different patient and technical factors.^21^

Prior studies have noted a strong interaction of tumor size, location, tip, and KI‐67 with bladder cancer staging and prognosis.^22^, ^23^, ^24^ This group's study of 134 lesions further confirmed that BC muscle invasion was associated with tumor size, stalk, base, ADC value, nADC value, and Ki‐67 LI. The wider the base, without a stalk, the longer the maximum diameter, the lower the aADC value and naADC value, and the higher the Ki67 LI, the more likely the tumor would invade the muscular layer. Regression analysis showed that ADC value (OR 0.009–0.014), nADC value (OR <0.001), maximum diameter (OR 2–2.156), and stalk (OR 14.741–26.010) were independently associated with muscle invasion. The bladder fundus (OR 7.829–12.215) or involvement of multiple sites (OR 21.887–37.506) were independently associated with muscle invasion compared to the bladder body. Some BC lesions have stalk‐like structures that extend from the bladder wall to the interior of the lesion. The pathological basis is fibrous tissue, capillaries, edema, and micro inflammatory cell infiltration.^25^ In contrast, stalkless BC was often an aggregate of tumor cells and was therefore more likely to invade the muscular layer. Kobayashi studied 132 patients with BC and found a correlation between lower ADC values and stalkless tumors.^26^ The median maximum diameter of the MIBC group was 3.6 cm, while the median maximum diameter of the NMIBC group was 1.5 cm, which was statistically different. The larger the tumor, the more likely it was to be spatially heterogeneous, exhibit variable biological behavior, and have an increased chance of invading the muscular layer. In our study, the risk of infiltration of the muscular layer was significantly higher in the wide base than in the narrow base (OR: 1.440–2.219, *p* > 0.05), which may be related to its maximum diameter and stalk. Some studies suggest that the larger the maximum diameter of the tumor, the wider its base, and that stalkless tumors have a wider base than stalked tumors.^27^

BC can occur in all parts of the bladder. And the 134 lesions in our group occurred mainly in the bladder fundus (47.46%), the bladder body (38.14%), and rarely the bladder apex and neck. NMIBC was more common in the bladder's body, apex, and neck than MIBC, and MIBC was more likely to occur in the fundus. The main reason was that the bladder fundus lacked muscularis mucosae, and the mucosa was closely linked to the muscular propria.^28^ When the bladder was empty or full, the mucosa remained smoaroth and did not form folds. Once BC grew more profound, it was easy to form muscular infiltration. If a single lesion involved multiple anatomical sites simultaneously, the probability of muscle infiltration would be significantly higher. In our study, 13 cases involved multiple sites, including 11 cases of MIBC. Of the 11 MIBC‐involved sites, six involved the bladder floor, four involved the bladder apex and body, and one lesion involved the bladder body and neck. Preoperative ADC or nADC combined with BC lesion morphology and site could predict the invasiveness and pathological stage of bladder cancer to a certain extent while considering the lesion qualification, which had high guiding significance for the clinical treatment plan.

As a proliferating cell‐associated nuclear antigen, Ki‐67 LI had a function closely related to mitosis, which was indispensable in cell proliferation. Therefore, in our study, it was suggested that higher Ki67 LI was riskier for BC myofibroblast infiltration, and it was moderately negatively correlated with ADC values and nADC with correlation coefficients of 0.604–0.634, respectively, similar to the findings of Kobayashi S et al. (*r* = −0.57, *p* < 0.001).^26^

Our study had some shortcomings: (a) This study was a single‐center retrospective study, and there was some bias in the included data, which needed further validation by multi‐center and large samples. (b) The ROIs were manually sketched, and there were some biases. (c) Due to the short follow‐up period, clinical issues related to the efficacy of tumor treatment and postoperative recurrence rate had not been explored.

## 5 CONCLUSIONS

This is the first report in which we found 3.0‐T MRI multi‐directional DWI combined with T2WI can be used for preoperative staging of BC, especially in the muscle layer invasion (stage T2 or higher) and non‐muscle layer invasion (stage T1 or lower). When the ADC and nADC values are lower, the maximum diameter is longer; the tumor is more likely to invade the muscle layer with no stalk and a wide base. In addition, TADC value and nTADC value help improve its diagnostic efficiency, and there is a negative correlation between ADC value and nADC and Ki‐67 LI. The method without MRI gadolinium contrast is easy to use in patients with renal insufficiency and has multiple reviews. It avoids the risk of contrast complications and is conducive to clinical preoperative imaging assessment and postoperative recurrence risk monitoring.

## AUTHOR CONTRIBUTIONS

**Wei Zhang:** Data curation (equal); formal analysis (equal); methodology (equal). **Zhichao Zhang:** Investigation (equal); supervision (equal); writing – original draft (equal). **Weixiong Xiao:** Formal analysis (equal); investigation (equal); methodology (equal); software (equal). **Yiqian Wang:** Formal analysis (equal); investigation (equal); methodology (equal). **Liefu Ye:** Software (equal); visualization (equal). **Yongbao Wei:** Conceptualization (equal); data curation (equal); funding acquisition (equal); writing – review and editing (equal). **Min Luo:** Conceptualization (equal); data curation (equal); supervision (equal).

## FUNDING INFORMATION

This study was supported by the China Urological Oncology Research Fund (027), the Middle‐aged Backbone Project Health and Family Planning Commission (2020GGB052; 2017‐ZQN‐13), and Fujian Natural Science Foundation (2021J05177; 2021J01359).

## CONFLICT OF INTEREST STATEMENT

The author has no conflict of interest to declare.

## ETHICS APPROVAL AND CONSENT TO PARTICIPATE

The study was conducted by the Declaration of Helsinki, and written informed consents were obtained from the guardians of these patients. The study was approved by the Ethics Committee of Fujian Provincial Hospital (K2020‐03‐115).

## Supporting information


Figure S1:

Table S1:

## Data Availability

All the data can be accessed from Min Luo (luomin6668@163.com).
