# Supplementary material for: Multiple directional DWI combined with T2WI in predicting muscle layer and Ki‐67 correlation in bladder cancer in 3.0‐T MRI
Source: Cancer Med. 2023 Mar 14;12(9):10462–72. doi: 10.1002/cam4.5782 (PMC10225208; doi:10.1002/cam4.5782)
Supplement: Supplementary file 1 — Figure S1: [file CAM4-12-10462-s001.docx]

A


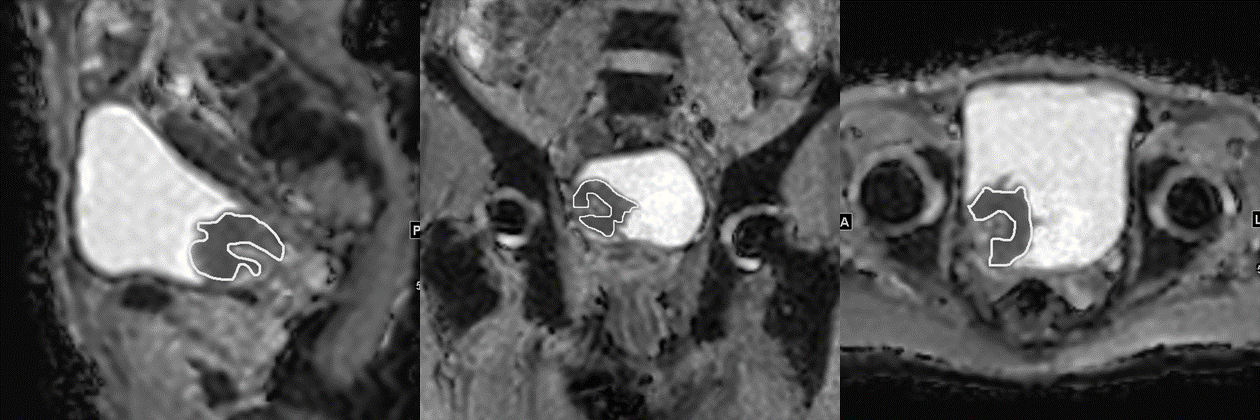


a

b

c

B


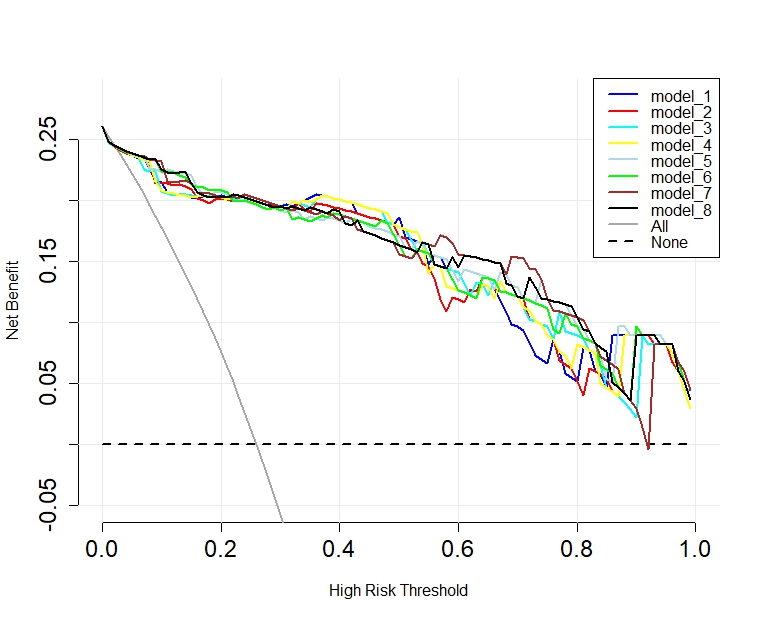


Supplementary Figure 1. Decision curve analysis of predictive models for MIBC detection. Multi-directional ADC image and region of interest (ROI) of the lesion (A). Net benefit is plotted against various threshold probabilities. Decision curves for each prediction model are shown (B).
