# Supplementary material for: Multiple directional DWI combined with T2WI in predicting muscle layer and Ki‐67 correlation in bladder cancer in 3.0‐T MRI
Source: Cancer Med. 2023 Mar 14;12(9):10462–72. doi: 10.1002/cam4.5782 (PMC10225208; doi:10.1002/cam4.5782)
Supplement: Supplementary file 2 — Table S1: [file CAM4-12-10462-s002.docx]

Supplementary Table 1 Bladder magnetic resonance imaging parameters.

|  | FOV(mm) | Matrix size | TR/TE(ms) | ST/SP(mm) | Rbw(Hz/pixel) | Acquisition time |
| --- | --- | --- | --- | --- | --- | --- |
| T2WI | 240×240 | 275×320 | 4820/85 | 3.5/0.4 | 2000 | 01:21/01:25/02:16 |
| DWI | 380×308 | 128×104 | 3700/48 | 3.5/0.4 | 2442 | 01:31/01:38/02:01 |

Note: According to multiple positioning, the sagittal position, oblique transverse axis position and oblique coronal position of BC were collected. The maximal axial position or oblique coronal position is parallel or perpendicular to the base of the tumor. Acquisition time of T2WI in transverse position was 01:21, sagittal position was 01:25, and coronal position was 02:16. Acquisition time of DWI in transverse position was 01:31, sagittal position was 01:38, and coronal position was 02:01.
